# Bis{(*E*)-2,4-diiodo-6-[(2-morpholinoeth­yl)imino­meth­yl]phenolato}nickel(II)

**DOI:** 10.1107/S1600536808015389

**Published:** 2008-05-30

**Authors:** Dong-Sheng Xia, Wu Chen, Hao Wang, Qing-Fu Zeng

**Affiliations:** a Engineering Research Center for the Clean Production of Textile Printing, Ministry of Education, Wuhan University of Science and Engineering, Wuhan 430073, People’s Republic of China

## Abstract

In the title mononuclear nickel(II) complex, [Ni(C_13_H_15_I_2_N_2_O_2_)_2_], the Ni^II^ atom is four-coordinated in a tetra­hedral geometry by the imine N and phenolate O atoms of the two Schiff base ligands. The O and N atoms of the morpholine substituent in the ligand are not involved in coordination to the Ni atom.

## Related literature

For related structures, see: Cheng *et al.* (2007 ▶); Li *et al.* (2007 ▶); Qiu *et al.* (2006 ▶); Shi *et al.* (2007 ▶); Wang *et al.* (2005 ▶); Zhu *et al.* (2003 ▶).
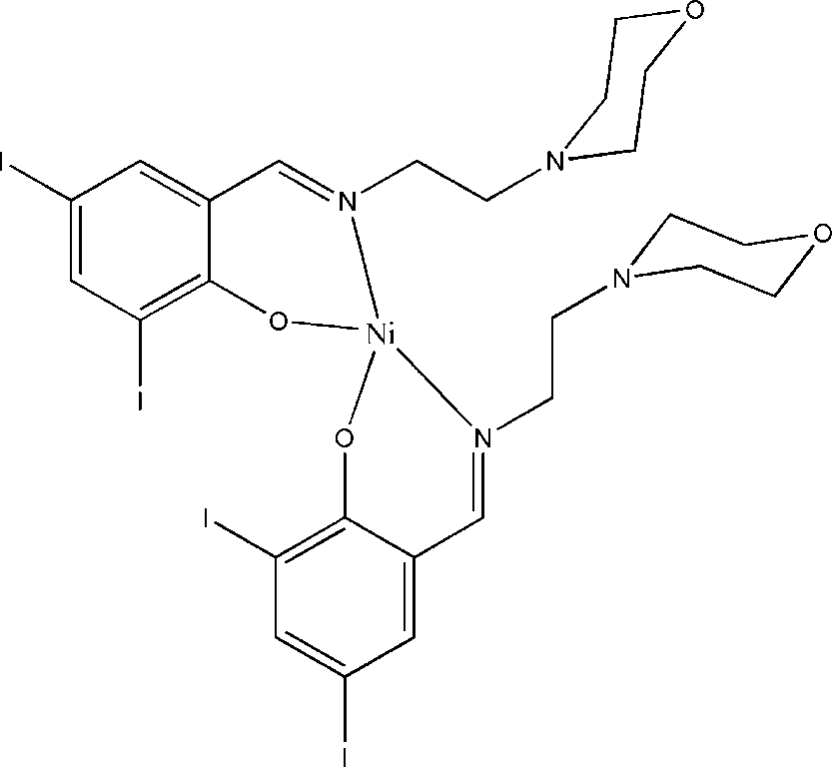


## Experimental

### 

#### Crystal data

[Ni(C_13_H_15_I_2_N_2_O_2_)_2_]
*M* *_r_* = 1028.85
Triclinic, 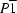

*a* = 9.940 (2) Å
*b* = 11.371 (2) Å
*c* = 14.526 (3) Å
α = 87.138 (3)°
β = 79.028 (4)°
γ = 76.197 (4)°
*V* = 1565.3 (5) Å^3^
*Z* = 2
Mo *K*α radiation
μ = 4.60 mm^−1^
*T* = 298 (2) K
0.17 × 0.15 × 0.15 mm

#### Data collection

Enraf–Nonius CAD-4 diffractometer
Absorption correction: ψ scan (North *et al.*, 1968 ▶) *T*_min_ = 0.465, *T*_max_ = 0.507
6131 measured reflections
6081 independent reflections
4486 reflections with *I* > 2σ(*I*)
*R*_int_ = 0.039

#### Refinement

*R*[*F* ^2^ > 2σ(*F* ^2^)] = 0.053
*wR*(*F* ^2^) = 0.154
*S* = 1.07
6081 reflections
340 parameters
H-atom parameters constrained
Δρ_max_ = 1.01 e Å^−3^
Δρ_min_ = −1.19 e Å^−3^

### 

Data collection: *CAD-4 Software* (Enraf–Nonius, 1989 ▶); cell refinement: *CAD-4 Software*; data reduction: *XCAD4* (Harms & Wocadlo, 1995 ▶); program(s) used to solve structure: *SHELXS97* (Sheldrick, 2008 ▶); program(s) used to refine structure: *SHELXL97* (Sheldrick, 2008 ▶); molecular graphics: *SHELXTL* (Sheldrick, 2008 ▶); software used to prepare material for publication: *SHELXTL*.

## Supplementary Material

Crystal structure: contains datablocks global, I. DOI: 10.1107/S1600536808015389/sj2504sup1.cif

Structure factors: contains datablocks I. DOI: 10.1107/S1600536808015389/sj2504Isup2.hkl

Additional supplementary materials:  crystallographic information; 3D view; checkCIF report

## Figures and Tables

**Table:** 

Ni—O4	1.956 (6)
Ni—O2	1.989 (6)
Ni—N2	2.001 (7)
Ni—N4	2.004 (7)

**Table:** 

O4—Ni—O2	104.7 (3)
O4—Ni—N2	102.8 (3)
O2—Ni—N2	93.7 (3)
O4—Ni—N4	94.2 (3)
O2—Ni—N4	101.5 (3)
N2—Ni—N4	153.5 (3)

**Fig. 1.**
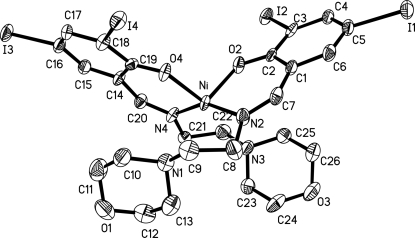
The structure of (I) showing 30% probability displacement ellipsoids and the atom-numbering scheme.

## supplementary crystallographic information

### Comment

As part of our ongoing interest in the structure of nickel(II) complexes (Zhu *et al.*, 2003), we report herein the crystal structure of the title compound, a new mononuclear nickel(II) complex, (I), Fig. 1, derived from the Schiff base ligand 2,4-diiodo-6-[(2-morpholin-4-ylethylimino)methyl]phenol.

The Ni^II^ atom in (I) is four-coordinate in a tetrahedral geometry, binding to the imine N and phenolate O atoms of the two Schiff base ligands. The O and N atoms of the morpholine substituent in the ligand lie well away from the coordination sphere of the Ni atom. The coordinate bond values (Table 1) are comparable to values observed in other similar nickel(II) complexes (Shi *et al.*, 2007; Li *et al.*, 2007; Cheng *et al.*, 2007; Qiu *et al.*, 2006; Wang *et al.*, 2005).

### Experimental

3,5-Diiodosalicylaldehyde (74.8 mg, 0.2 mmol), 2-morpholin-4-ylethylamine (26.0 mg, 0.2 mmol), and NiCl_2_.6H_2_O (23.8 mg, 0.1 mmol) were dissolved in methanol (30 ml). The mixture was stirred for 30 min at room temperature. The resulting solution was left in air for a few days, yielding green crystals.

### Refinement

H atoms were placed in idealized positions and constrained to ride on their parent atoms with C–H distances in the range 0.93–0.97 Å, and with *U*_iso_(H) set at 1.2*U*_eq_(C).

### Crystal data

**Table:**

[Ni(C_13_H_15_I_2_N_2_O_2_)_2_]	*Z* = 2
*M**_r_* = 1028.85	*F*_000_ = 972
Triclinic, *P*1	*D*_x_ = 2.183 Mg m^−^^3^
Hall symbol: -P 1	Mo *K*α radiation λ = 0.71073 Å
*a* = 9.940 (2) Å	Cell parameters from 3273 reflections
*b* = 11.371 (2) Å	θ = 2.4–25.3º
*c* = 14.526 (3) Å	µ = 4.60 mm^−^^1^
α = 87.138 (3)º	*T* = 298 (2) K
β = 79.028 (4)º	Block, green
γ = 76.197 (4)º	0.17 × 0.15 × 0.15 mm
*V* = 1565.3 (5) Å^3^	

### Data collection

**Table:**

Enraf–Nonius CAD-4 diffractometer	6081 independent reflections
Radiation source: fine-focus sealed tube	4486 reflections with *I* > 2σ(*I*)
Monochromator: graphite	*R*_int_ = 0.039
*T* = 298(2) K	θ_max_ = 26.0º
ω/2θ scans	θ_min_ = 1.4º
Absorption correction: psi scan(North *et al.*, 1968)	*h* = −11→12
*T*_min_ = 0.465, *T*_max_ = 0.507	*k* = −13→14
6131 measured reflections	*l* = −16→17

### Refinement

**Table:**

Refinement on *F*^2^	Secondary atom site location: difference Fourier map
Least-squares matrix: full	Hydrogen site location: inferred from neighbouring sites
*R*[*F*^2^ > 2σ(*F*^2^)] = 0.053	H-atom parameters constrained
*wR*(*F*^2^) = 0.154	*w* = 1/[σ^2^(*F*_o_^2^) + (0.0708*P*)^2^ + 8.6966*P*] where *P* = (*F*_o_^2^ + 2*F*_c_^2^)/3
*S* = 1.07	(Δ/σ)_max_ = 0.001
6081 reflections	Δρ_max_ = 1.01 e Å^−^^3^
340 parameters	Δρ_min_ = −1.19 e Å^−^^3^
Primary atom site location: structure-invariant direct methods	Extinction correction: none

### Special details

**Table:**

Geometry. All esds (except the esd in the dihedral angle between two l.s. planes) are estimated using the full covariance matrix. The cell esds are taken into account individually in the estimation of esds in distances, angles and torsion angles; correlations between esds in cell parameters are only used when they are defined by crystal symmetry. An approximate (isotropic) treatment of cell esds is used for estimating esds involving l.s. planes.
Refinement. Refinement of F^2^ against ALL reflections. The weighted R-factor wR and goodness of fit S are based on F^2^, conventional R-factors R are based on F, with F set to zero for negative F^2^. The threshold expression of F^2^ > 2sigma(F^2^) is used only for calculating R-factors(gt) etc. and is not relevant to the choice of reflections for refinement. R-factors based on F^2^ are statistically about twice as large as those based on F, and R- factors based on ALL data will be even larger.

### Fractional atomic coordinates and isotropic or equivalent isotropic displacement parameters (Å^2^)

**Table:**

	*x*	*y*	*z*	*U*_iso_*/*U*_eq_	
Ni	0.67459 (12)	0.74611 (9)	0.74968 (7)	0.0370 (3)	
I1	1.03986 (8)	0.21944 (6)	1.02656 (5)	0.0587 (2)	
I2	1.08919 (7)	0.41240 (6)	0.63145 (5)	0.0566 (2)	
I3	0.89016 (8)	1.25985 (6)	0.44905 (5)	0.0581 (2)	
I4	0.86000 (8)	1.08336 (6)	0.84580 (5)	0.0544 (2)	
O1	0.2528 (9)	1.0772 (9)	0.6949 (6)	0.088 (3)	
O2	0.8403 (7)	0.6056 (5)	0.7347 (4)	0.0476 (15)	
O3	0.3977 (9)	0.4099 (8)	0.8193 (6)	0.083 (3)	
O4	0.7534 (7)	0.8868 (5)	0.7567 (4)	0.0468 (15)	
N1	0.4262 (9)	0.9357 (7)	0.8185 (5)	0.0509 (19)	
N2	0.6094 (8)	0.7093 (7)	0.8847 (5)	0.0479 (18)	
N3	0.5530 (8)	0.5511 (7)	0.6897 (5)	0.0477 (18)	
N4	0.6484 (8)	0.7785 (6)	0.6166 (5)	0.0416 (16)	
C1	0.8091 (9)	0.5352 (7)	0.8924 (6)	0.0405 (19)	
C2	0.8775 (9)	0.5295 (7)	0.7959 (6)	0.0363 (18)	
C3	0.9936 (9)	0.4299 (8)	0.7732 (6)	0.043 (2)	
C4	1.0432 (9)	0.3416 (8)	0.8368 (6)	0.043 (2)	
H4	1.1195	0.2774	0.8170	0.051*	
C5	0.9773 (10)	0.3513 (8)	0.9290 (7)	0.046 (2)	
C6	0.8621 (10)	0.4491 (8)	0.9557 (6)	0.048 (2)	
H6	0.8193	0.4566	1.0186	0.057*	
C7	0.6868 (10)	0.6243 (8)	0.9306 (6)	0.046 (2)	
H7	0.6579	0.6225	0.9953	0.055*	
C8	0.4785 (12)	0.7862 (10)	0.9417 (7)	0.064 (3)	
H8A	0.3971	0.7572	0.9340	0.077*	
H8B	0.4855	0.7793	1.0076	0.077*	
C9	0.4594 (12)	0.9162 (10)	0.9117 (7)	0.061 (3)	
H9A	0.3840	0.9656	0.9560	0.073*	
H9B	0.5452	0.9421	0.9130	0.073*	
C10	0.4274 (12)	1.0621 (10)	0.7914 (9)	0.068 (3)	
H10A	0.5204	1.0751	0.7912	0.081*	
H10B	0.3608	1.1159	0.8376	0.081*	
C11	0.3903 (16)	1.0927 (13)	0.6976 (10)	0.092 (4)	
H11A	0.3938	1.1761	0.6823	0.111*	
H11B	0.4588	1.0412	0.6509	0.111*	
C12	0.2531 (14)	0.9555 (13)	0.7185 (9)	0.080 (4)	
H12A	0.3235	0.9036	0.6730	0.096*	
H12B	0.1620	0.9413	0.7145	0.096*	
C13	0.2827 (13)	0.9214 (11)	0.8133 (7)	0.067 (3)	
H13A	0.2774	0.8380	0.8267	0.080*	
H13B	0.2130	0.9726	0.8597	0.080*	
C14	0.7537 (9)	0.9542 (8)	0.5987 (6)	0.0408 (19)	
C15	0.7930 (9)	1.0375 (8)	0.5305 (7)	0.044 (2)	
H15	0.7818	1.0294	0.4692	0.053*	
C16	0.8466 (10)	1.1292 (8)	0.5514 (7)	0.045 (2)	
C17	0.8658 (9)	1.1437 (8)	0.6402 (6)	0.043 (2)	
H17	0.9010	1.2079	0.6546	0.052*	
C18	0.8319 (10)	1.0611 (8)	0.7084 (6)	0.045 (2)	
C19	0.7810 (8)	0.9604 (7)	0.6922 (6)	0.0364 (18)	
C20	0.6925 (10)	0.8632 (8)	0.5680 (6)	0.043 (2)	
H20	0.6841	0.8668	0.5052	0.052*	
C21	0.5820 (9)	0.7051 (8)	0.5668 (6)	0.043	
H21A	0.6121	0.7132	0.4997	0.051*	
H21B	0.4804	0.7344	0.5813	0.051*	
C22	0.6216 (11)	0.5733 (10)	0.5948 (7)	0.061	
H22A	0.5954	0.5246	0.5513	0.074*	
H22B	0.7230	0.5485	0.5904	0.074*	
C23	0.4055 (11)	0.5588 (11)	0.6960 (8)	0.064 (3)	
H23A	0.3925	0.5025	0.6523	0.077*	
H23B	0.3591	0.6400	0.6791	0.077*	
C24	0.3403 (14)	0.5295 (13)	0.7941 (9)	0.078 (4)	
H24A	0.3558	0.5846	0.8378	0.094*	
H24B	0.2394	0.5411	0.7985	0.094*	
C25	0.6143 (11)	0.4272 (9)	0.7168 (8)	0.061 (3)	
H25A	0.7143	0.4179	0.7152	0.073*	
H25B	0.6038	0.3713	0.6717	0.073*	
C26	0.5451 (14)	0.3955 (12)	0.8137 (10)	0.081 (4)	
H26A	0.5873	0.3124	0.8286	0.098*	
H26B	0.5612	0.4475	0.8595	0.098*	

### Atomic displacement parameters (Å^2^)

**Table:**

	*U*^11^	*U*^22^	*U*^33^	*U*^12^	*U*^13^	*U*^23^
Ni	0.0507 (6)	0.0302 (5)	0.0320 (5)	−0.0192 (5)	−0.0025 (5)	0.0092 (4)
I1	0.0742 (5)	0.0474 (4)	0.0577 (4)	−0.0181 (3)	−0.0193 (3)	0.0167 (3)
I2	0.0634 (4)	0.0504 (4)	0.0501 (4)	−0.0206 (3)	0.0121 (3)	0.0055 (3)
I3	0.0732 (5)	0.0503 (4)	0.0565 (4)	−0.0323 (3)	−0.0088 (3)	0.0215 (3)
I4	0.0700 (4)	0.0545 (4)	0.0467 (4)	−0.0290 (3)	−0.0126 (3)	0.0029 (3)
O1	0.075 (6)	0.094 (7)	0.072 (6)	0.014 (5)	−0.004 (5)	0.017 (5)
O2	0.061 (4)	0.038 (3)	0.040 (3)	−0.014 (3)	0.000 (3)	0.009 (3)
O3	0.084 (6)	0.088 (6)	0.081 (6)	−0.049 (5)	0.003 (5)	0.021 (5)
O4	0.067 (4)	0.035 (3)	0.040 (3)	−0.024 (3)	0.000 (3)	0.008 (3)
N1	0.059 (5)	0.050 (5)	0.041 (4)	−0.012 (4)	−0.001 (4)	−0.009 (4)
N2	0.054 (5)	0.041 (4)	0.040 (4)	−0.005 (3)	0.002 (3)	0.007 (3)
N3	0.052 (5)	0.048 (4)	0.046 (4)	−0.022 (4)	−0.006 (4)	0.010 (3)
N4	0.053 (4)	0.037 (4)	0.037 (4)	−0.025 (3)	0.004 (3)	−0.001 (3)
C1	0.046 (5)	0.032 (4)	0.043 (5)	−0.014 (4)	−0.003 (4)	0.007 (4)
C2	0.046 (5)	0.033 (4)	0.039 (4)	−0.025 (4)	−0.011 (4)	0.010 (3)
C3	0.049 (5)	0.036 (4)	0.046 (5)	−0.027 (4)	0.005 (4)	0.004 (4)
C4	0.044 (5)	0.035 (4)	0.046 (5)	−0.011 (4)	−0.002 (4)	0.009 (4)
C5	0.060 (6)	0.034 (5)	0.050 (5)	−0.019 (4)	−0.017 (4)	0.012 (4)
C6	0.060 (6)	0.051 (5)	0.034 (5)	−0.022 (5)	−0.008 (4)	0.012 (4)
C7	0.064 (6)	0.047 (5)	0.029 (4)	−0.022 (5)	−0.003 (4)	0.008 (4)
C8	0.069 (7)	0.060 (6)	0.045 (6)	0.006 (5)	0.006 (5)	0.006 (5)
C9	0.071 (7)	0.061 (6)	0.047 (6)	−0.011 (5)	−0.003 (5)	−0.018 (5)
C10	0.064 (7)	0.052 (6)	0.080 (8)	−0.017 (5)	0.010 (6)	−0.007 (6)
C11	0.097 (11)	0.076 (9)	0.086 (10)	−0.017 (8)	0.021 (8)	0.012 (7)
C12	0.074 (8)	0.087 (9)	0.076 (8)	−0.010 (7)	−0.016 (7)	−0.021 (7)
C13	0.086 (8)	0.074 (8)	0.042 (6)	−0.030 (6)	−0.001 (5)	−0.003 (5)
C14	0.051 (5)	0.040 (5)	0.037 (4)	−0.026 (4)	−0.005 (4)	0.008 (4)
C15	0.045 (5)	0.037 (5)	0.048 (5)	−0.011 (4)	0.000 (4)	0.000 (4)
C16	0.045 (5)	0.034 (4)	0.052 (5)	−0.013 (4)	0.006 (4)	0.004 (4)
C17	0.049 (5)	0.031 (4)	0.050 (5)	−0.015 (4)	−0.010 (4)	0.011 (4)
C18	0.055 (5)	0.040 (5)	0.040 (5)	−0.020 (4)	0.001 (4)	−0.002 (4)
C19	0.028 (4)	0.036 (4)	0.044 (5)	−0.011 (3)	−0.001 (3)	0.002 (4)
C20	0.056 (5)	0.048 (5)	0.032 (4)	−0.022 (4)	−0.013 (4)	0.009 (4)
C21	0.043	0.043	0.043	−0.010	−0.008	0.000
C22	0.061	0.061	0.061	−0.014	−0.011	0.000
C23	0.059 (6)	0.072 (7)	0.057 (6)	−0.015 (5)	−0.009 (5)	0.023 (6)
C24	0.079 (8)	0.101 (10)	0.063 (7)	−0.055 (8)	0.008 (6)	0.008 (7)
C25	0.058 (6)	0.051 (6)	0.080 (8)	−0.020 (5)	−0.019 (6)	0.006 (5)
C26	0.086 (9)	0.068 (8)	0.095 (10)	−0.034 (7)	−0.017 (7)	0.029 (7)

### Geometric parameters (Å, °)

**Table:**

Ni—O4	1.956 (6)	C9—H9A	0.9700
Ni—O2	1.989 (6)	C9—H9B	0.9700
Ni—N2	2.001 (7)	C10—C11	1.483 (18)
Ni—N4	2.004 (7)	C10—H10A	0.9700
I1—C5	2.079 (8)	C10—H10B	0.9700
I2—C3	2.095 (9)	C11—H11A	0.9700
I3—C16	2.108 (8)	C11—H11B	0.9700
I4—C18	2.103 (9)	C12—C13	1.477 (16)
O1—C12	1.408 (16)	C12—H12A	0.9700
O1—C11	1.427 (17)	C12—H12B	0.9700
O2—C2	1.254 (9)	C13—H13A	0.9700
O3—C24	1.403 (15)	C13—H13B	0.9700
O3—C26	1.422 (15)	C14—C15	1.403 (11)
O4—C19	1.260 (10)	C14—C19	1.442 (12)
N1—C9	1.448 (13)	C14—C20	1.446 (12)
N1—C10	1.473 (13)	C15—C16	1.349 (13)
N1—C13	1.490 (14)	C15—H15	0.9300
N2—C7	1.322 (11)	C16—C17	1.363 (13)
N2—C8	1.504 (12)	C17—C18	1.382 (11)
N3—C23	1.433 (13)	C17—H17	0.9300
N3—C22	1.456 (13)	C18—C19	1.403 (12)
N3—C25	1.462 (12)	C20—H20	0.9300
N4—C20	1.278 (10)	C21—C22	1.511 (13)
N4—C21	1.469 (11)	C21—H21A	0.9700
C1—C6	1.390 (12)	C21—H21B	0.9700
C1—C7	1.422 (13)	C22—H22A	0.9700
C1—C2	1.435 (12)	C22—H22B	0.9700
C2—C3	1.411 (12)	C23—C24	1.506 (14)
C3—C4	1.396 (12)	C23—H23A	0.9700
C4—C5	1.372 (13)	C23—H23B	0.9700
C4—H4	0.9300	C24—H24A	0.9700
C5—C6	1.401 (13)	C24—H24B	0.9700
C6—H6	0.9300	C25—C26	1.512 (16)
C7—H7	0.9300	C25—H25A	0.9700
C8—C9	1.499 (15)	C25—H25B	0.9700
C8—H8A	0.9700	C26—H26A	0.9700
C8—H8B	0.9700	C26—H26B	0.9700
			
O4—Ni—O2	104.7 (3)	O1—C12—H12A	108.9
O4—Ni—N2	102.8 (3)	C13—C12—H12A	108.9
O2—Ni—N2	93.7 (3)	O1—C12—H12B	108.9
O4—Ni—N4	94.2 (3)	C13—C12—H12B	108.9
O2—Ni—N4	101.5 (3)	H12A—C12—H12B	107.7
N2—Ni—N4	153.5 (3)	C12—C13—N1	109.3 (9)
C12—O1—C11	107.0 (9)	C12—C13—H13A	109.8
C2—O2—Ni	127.5 (6)	N1—C13—H13A	109.8
C24—O3—C26	108.3 (9)	C12—C13—H13B	109.8
C19—O4—Ni	127.9 (6)	N1—C13—H13B	109.8
C9—N1—C10	107.9 (9)	H13A—C13—H13B	108.3
C9—N1—C13	113.4 (8)	C15—C14—C19	119.4 (8)
C10—N1—C13	106.0 (8)	C15—C14—C20	116.3 (8)
C7—N2—C8	116.5 (8)	C19—C14—C20	124.2 (7)
C7—N2—Ni	121.5 (6)	C16—C15—C14	121.8 (9)
C8—N2—Ni	121.6 (6)	C16—C15—H15	119.1
C23—N3—C22	112.1 (8)	C14—C15—H15	119.1
C23—N3—C25	106.1 (8)	C15—C16—C17	120.9 (8)
C22—N3—C25	108.7 (8)	C15—C16—I3	120.5 (7)
C20—N4—C21	115.2 (7)	C17—C16—I3	118.5 (6)
C20—N4—Ni	121.8 (6)	C16—C17—C18	118.7 (8)
C21—N4—Ni	123.0 (5)	C16—C17—H17	120.6
C6—C1—C7	115.4 (8)	C18—C17—H17	120.6
C6—C1—C2	119.8 (8)	C17—C18—C19	124.2 (8)
C7—C1—C2	124.8 (7)	C17—C18—I4	119.0 (7)
O2—C2—C3	121.0 (8)	C19—C18—I4	116.8 (6)
O2—C2—C1	124.2 (8)	O4—C19—C18	121.7 (8)
C3—C2—C1	114.8 (7)	O4—C19—C14	123.5 (7)
C4—C3—C2	125.0 (8)	C18—C19—C14	114.6 (7)
C4—C3—I2	119.0 (7)	N4—C20—C14	128.0 (8)
C2—C3—I2	116.0 (6)	N4—C20—H20	116.0
C5—C4—C3	118.8 (8)	C14—C20—H20	116.0
C5—C4—H4	120.6	N4—C21—C22	110.7 (8)
C3—C4—H4	120.6	N4—C21—H21A	109.5
C4—C5—C6	118.8 (8)	C22—C21—H21A	109.5
C4—C5—I1	120.9 (7)	N4—C21—H21B	109.5
C6—C5—I1	120.2 (7)	C22—C21—H21B	109.5
C1—C6—C5	122.9 (8)	H21A—C21—H21B	108.1
C1—C6—H6	118.6	N3—C22—C21	112.3 (8)
C5—C6—H6	118.6	N3—C22—H22A	109.2
N2—C7—C1	127.5 (8)	C21—C22—H22A	109.2
N2—C7—H7	116.3	N3—C22—H22B	109.2
C1—C7—H7	116.3	C21—C22—H22B	109.2
C9—C8—N2	110.8 (8)	H22A—C22—H22B	107.9
C9—C8—H8A	109.5	N3—C23—C24	110.3 (9)
N2—C8—H8A	109.5	N3—C23—H23A	109.6
C9—C8—H8B	109.5	C24—C23—H23A	109.6
N2—C8—H8B	109.5	N3—C23—H23B	109.6
H8A—C8—H8B	108.1	C24—C23—H23B	109.6
N1—C9—C8	112.4 (9)	H23A—C23—H23B	108.1
N1—C9—H9A	109.1	O3—C24—C23	111.6 (11)
C8—C9—H9A	109.1	O3—C24—H24A	109.3
N1—C9—H9B	109.1	C23—C24—H24A	109.3
C8—C9—H9B	109.1	O3—C24—H24B	109.3
H9A—C9—H9B	107.9	C23—C24—H24B	109.3
N1—C10—C11	112.3 (10)	H24A—C24—H24B	108.0
N1—C10—H10A	109.2	N3—C25—C26	111.9 (9)
C11—C10—H10A	109.2	N3—C25—H25A	109.2
N1—C10—H10B	109.2	C26—C25—H25A	109.2
C11—C10—H10B	109.2	N3—C25—H25B	109.2
H10A—C10—H10B	107.9	C26—C25—H25B	109.2
O1—C11—C10	111.2 (10)	H25A—C25—H25B	107.9
O1—C11—H11A	109.4	O3—C26—C25	111.0 (10)
C10—C11—H11A	109.4	O3—C26—H26A	109.4
O1—C11—H11B	109.4	C25—C26—H26A	109.4
C10—C11—H11B	109.4	O3—C26—H26B	109.4
H11A—C11—H11B	108.0	C25—C26—H26B	109.4
O1—C12—C13	113.6 (11)	H26A—C26—H26B	108.0
